# DUbbing Language-therapy CINEma-based in Aphasia post-Stroke (DULCINEA): study protocol for a randomized crossover pilot trial

**DOI:** 10.1186/s13063-021-05956-5

**Published:** 2022-01-06

**Authors:** Blanca Fuentes, Lydia de la Fuente-Gómez, Cristian Sempere-Iborra, Celia Delgado-Fernández, Aida Tarifa-Rodríguez, María Alonso de Leciñana, Elena de Celis-Ruiz, Raquel Gutiérrez-Zúñiga, José López-Tàppero, Marta Martín Alonso, Sylvia Pastor-Yborra, Ricardo Rigual, Gerardo Ruiz-Ares, Jorge Rodríguez-Pardo, Javier Virués-Ortega, Alberto M. Borobia, Paloma Blanco, Nereida Bueno-Guerra

**Affiliations:** 1 Department of Neurology and Stroke Center, La Paz University Hospital, Department of Medicine, Neurosciences Unit, Universidad Autónoma de Madrid, IdiPAZ Institute for Health Research, Madrid, Spain; 2 Neurosciences Area, IdiPAZ Institute for Health Research, Madrid, Spain; 3 Department of Psychology, Universidad Autónoma de Madrid, Madrid, Spain; 4 Speech and Language Unit, Department of Rehabilitation, La Paz University Hospital and Universidad Complutense de Madrid, Madrid, Spain; 5 Speech and Language Unit, Department of Rehabilitation, La Paz University Hospital, Madrid, Spain; 6 The University of Auckland, Auckland, New Zealand; 7 Clinical Trials Unit, Department of Clinical Pharmacology, La Paz University Hospital, IdiPAZ Institute for Health Research, Madrid, Spain; 8 Asociación Afasia Activa, Madrid, Spain; 9 Department of Psychology, Universidad Pontificia Comillas, Madrid, Spain

**Keywords:** Stroke, Aphasia, Randomized clinical trial, Protocol

## Abstract

**Background:**

Communication is one of the most important predictors of social reintegration after stroke. Approximately 15–42% of stroke survivors experience post-stroke aphasia. Helping people recover from aphasia is one of the research priorities after a stroke. Our aim is to develop and validate a new therapy integrating dubbing techniques to improve functional communication.

**Methods:**

The research project is structured as three work packages (WP). WP1: development of the dubbed language cinema-based therapy: Two research assistants (a speech therapist and a dubbing actor) will select the clips, mute specific words/sentences in progressive speech difficulty, and guide patients to dub them across sessions. Words to be dubbed will be those considered to be functionally meaningful by a representative sample of aphasic patients and relatives through an online survey. WP2: a randomized, crossover, interventional pilot study with the inclusion of 54 patients with post-stroke non-fluent aphasia. Patients will be treated individually in 40-min sessions twice per week for 8 weeks. Primary outcomes will be significant pre/post differences in scores in the Communicative Activity Log (CAL) questionnaire and Boston Diagnostic Aphasia Examination (BDAE) administered by a psychologist blinded to the patients’ clinical characteristics. Secondary outcomes: General Health Questionnaire (GHQ)-12, Stroke Aphasia Quality of Life Scale (SAQOL-39), Western Aphasia Battery Revised (WAB-R), and the Stroke Aphasic Depression Questionnaire (SADQ10). WP3: educational activities and dissemination of results. WP3 includes educational activities to improve public knowledge of aphasia and dissemination of the results, with the participation of the Spanish patients’ association *Afasia Activa*.

**Discussion:**

This pilot clinical trial will explore the efficacy of a new therapeutic tool based on dubbing techniques and computer technology to improve functional communication of patients suffering from post-stroke aphasia with the use of standardized test assessment.

**Trial registration:**

ClinicalTrials.govNCT04289493. Registered on 28 February 2020.

## Background

Stroke is a leading cause of disability, and the absolute number of people who develop stroke each year is growing worryingly, as are the number of survivors and the overall burden of stroke [1]. Communication is a vital aspect of daily functioning, being the most important predictor of social reintegration after a stroke [2]. It is estimated that approximately 15–42% of stroke survivors experience post-stroke aphasia, affecting some of the language modalities, including the production and understanding of speech, reading, and writing. Aphasia is associated not only with a greater risk of mortality after stroke, but also with long-term disability, greatly impacting the quality of life [3]. Hence, helping people recover from aphasia has been identified by consensus among survivors, caregivers, and health professionals as one of the top 10 research priorities relating to life after stroke [4]. Current international guidelines recommend speech and language therapy (SLT) for stroke survivors [5, 6]. However, according to a Cochrane meta-analysis, available SLT differ in therapy regimens (intensity, dosage, duration), delivery models (group, one-to-one, volunteer, computed-facilitated), approach, and outcome measurements [7]. Thus, there is no universally accepted SLT for post-stroke aphasia. Moreover, several SLTs use child-like materials, therefore not being generalizable for a daily adult life. This is one of the main challenges of intensive SLT since it produces high rates of early dropouts, up to 30% [7], as patients do not feel that their preferences are being covered.

Computer-based technology such as mobile apps, video games, and virtual reality is increasingly being used with success in post-stroke motor rehabilitation [8]. However, SLT has not embraced the same path [9]. To our knowledge, the first attempt at using audiovisual material for SLT purposes dates from the 60's. Researchers from the University of Chicago Speech and Language Clinic used film strips with various topics for homebound and outpatient aphasic patients. Patients showed higher engagement with therapy and increased their spontaneous self-expression [10]. Recent studies used videos in SLT for instructional or speech-triggering purposes [11, 12]. Results were positive, with an improvement in patients’ knowledge about aphasia and language skills; however, the scripts used were very limited and the patients reported their wish to have a wider and more varied range of words to practice. Besides, the use of word and sentence imitation in a computer-based intensive therapy improved the narrative production of 19 patients in a before and after trial [13]. Although a randomized clinical trial was designed to assess the effect of intensive imitation therapy (NCT00713050) [14], to our knowledge, no results other than non-randomized case series have been reported from that study [13].

We conducted a PubMed search with the terms (((computer-based) AND (therapy)) AND (stroke)) AND (aphasia), retrieving a total of 25 articles (last search conducted on October 30, 2021). Of these, only five presented the results of randomized clinical trials [15–19], and an additional completed clinical trial was found on the ClinicalTrials.gov website [20] (Table 1). Clearly, we need to change our therapeutic approach in post-stroke aphasia and take advantage of existing technology.

**Table 1:** Summary of completed computer-based clinical trials on post-stroke aphasia

Reference	Trial ID	Design	Patients	Interventions	Main results
Kesav P et al. *J Neurol Sci* 2017; 380:137–141 [15]	Clinical Trials registry India 2016/08/0120121	Prospective open randomized, controlled trial with blinded endpoint evaluation.	20	• Group A: less intensive (12 therapy sessions of conventional professional-based SLT) • Group B: more intensive (12 therapy sessions of conventional professional-based SLT + 12 h of computer-based SLT)	• Less intensive worked better, but authors still recommend using computer-based
Grechuta K et al. *Stroke* 2019; 50:1270–1274 [16]	NCT02928822	Randomized, controlled, parallel-group trial	17	• Control group (*N*=8): standard treatment • Experimental group (*N*=9): augmented embodied therapy with the Rehabilitation Gaming System for aphasia.	• Both groups significantly improved on the BDAE and on the lexical access-vocabulary test. • Only the Rehabilitation Gaming System for aphasia group improved on the CAL and showed therapy-induced improvements in language and communication at 16 weeks of follow-up.
Palmer R et al. *Lancet Neurol*. 2019; 18: 821–33 [17]	ISRCTN68798818	Pragmatic, superiority, three-arm, individually randomized, single-blind, parallel-group trial.	278	• Control group: 6 months of usual care (usual care group) • CSLT Group: Daily self-managed CSLT plus usual care • Attention control plus usual care: paper-based puzzle book activities (e.g., sudoku, spot the difference, word searches, or coloring) on a daily basis.	• CSLT plus usual care resulted in a clinically significant improvement in personally relevant word finding but did not result in an improvement in conversation.
Cherney LR et al. *Clin Rehabil* 2021; 35: 976–987 [18]	NCT04413136	Single-blind, randomized placebo-controlled trial	32	• Experimental treatment (*N*=19): Web ORLA (Oral Reading for Language in Aphasia) • Control group (*N*=13): a commercially available computer game. Both groups were instructed to practice 90 min/day, 6 days/week for 6 weeks.	• No significant difference in the gain from pre-treatment to post-treatment between groups. • The Web ORLA group showed significantly greater gains at the 6-week follow-up than the control group.
Spaccavento S et al. *J Communication Disorders* 2021:106158 [19]	NA	Pilot randomized non-inferiority study	22	• Experimental group: computer-based • Control group: therapist-mediated aphasia treatment Both groups received one 50-min session for 5 days per week over a period of 8 weeks.	• Participants in both groups improved in language skills, functional communication, and quality-of-life measures from pre- to post-treatment • No significant differences between groups.
Elhakeem ES et al. *The Egyptian J Otolaryngology* 2021; 37:77 [20]	NCT04717180	Randomized controlled trial with blinded endpoint evaluation	50	• Group I: 48 sessions using the Arabic software program • Group II: 48 sessions of conventional Therapy	• Significant improvement from the baseline in both groups. • No significant difference in post-therapy results between groups except for some secondary items, whereas group I showed more significant improvement (phrase length, melodic line, word-finding relative to fluency, paraphasia, repetition, responsive naming, Boston naming test)

*BDAE* Boston Diagnostic Aphasia Examination, *CAL* Communicative Activity Log, *CSLT* Computer-based Speech and Language Therapy, *NA* not available, *SLT* Speech and Language Therapy

Sources: https://clinicaltrials.gov [search terms (Post-stroke aphasia) AND (computer); filters: completed] and PubMED [(computer-based) AND (therapy) AND (stroke) AND (aphasia)]. Last search conducted on October 30, 2021

Factors underlying language recovery, such as visual cue integration, self-performance assessment, motor coordination, intonation, emotion, and motivation, are supported by the latest discoveries in neuroscience as separate components [21–24]. We think that including dubbing in post-stroke aphasia rehabilitation provision can link technology with language factor integration. Dubbing actors need to articulate words within a given emotional context by paying attention to an audiovisual input. In a preliminary work, we developed a single-patient open trial with dubbing techniques in a 71-year-old woman with 15 months of chronic global aphasia after having undergone classical speech therapy (i.e., mirror repetition) with two different speech therapists. Prior to the dubbing therapy, the patient could only pronounce some Spanish filler words and some short words if actively shaped and helped. After participating in only two dubbing sessions, she spontaneously incorporated the dubbed words in the proper contexts and increased the time of sustained attention as compared with the time of attention paid in previous SLT sessions [25]. In that single-case study, we excluded potential natural recovery because the acquisition of the targeted words occurred 2 years after the stroke, when the linguistic impairment was already chronic, and the patient had not incorporated other words different from those trained for during the pilot. Therefore, the linguistic improvement appears to be attributed to the experimental intervention.

Finally, trials on post-stroke aphasia can benefit from collaboration between patients, clinicians, researchers, and the use of standardized outcome measures [7]. Our research project counts on the collaboration of post-stroke aphasic patients, who are actively consulted to choose the most useful training material, and we use the standardized tests to assess our results.

## Methods

Our aim is to develop and validate a new therapy integrating dubbing techniques to improve functional communication. This research project is structured as three work packages (WP). WP1: development of the dubbed language cinema-based therapy; WP2: a randomized, crossover, interventional pilot study; WP3: educational activities to improve public knowledge of aphasia and dissemination of the results. Here we present the protocol corresponding to the WP2 (clinical trial).

### Design

A randomized, crossover, interventional pilot study, following the CONSORT guidelines on randomized pilot and feasibility studies [26] and the SPIRIT 2013 Recommendations for Interventional Trials [27].

### Patient population

The Departments of Neurology and Rehabilitation (Speech Therapy Unit) at La Paz University Hospital and “Afasia Activa”, a non-profit patients’ association, will help with recruitment.

- Inclusion criteria:
  - Non-fluent aphasia due to ischaemic stroke in the left hemisphere without neuroimaging evidence of lesions in the right hemisphere;
  - Standard program of conventional speech therapy previously completed;
  - Severely restricted language; poor repetition even for single words and moderately preserved language comprehension (i.e., not exceeding the 70th percentile in Repetition Scale plus exceeding the 15th percentile in Listening Comprehension Scale as an average score in word comprehension and command subscales and complex ideational material) in the Boston Diagnostic Aphasia Examination (BDAE);
  - Signed informed consent. All the patients, or their guardian or legal representative, will be asked to sign a written informed consent form after a detailed explanation of the nature and purpose of this study and before undergoing any of the procedures related to the clinical trial. An aphasia-friendly information sheet containing large text and simplified language will be provided.
- Exclusion criteria:
  - Any clinical condition or other characteristics that precluded appropriate follow-up;
  - Simultaneous participation in any therapeutic trial assessing post-stroke recovery.

### Randomization

**Fig. 1:**
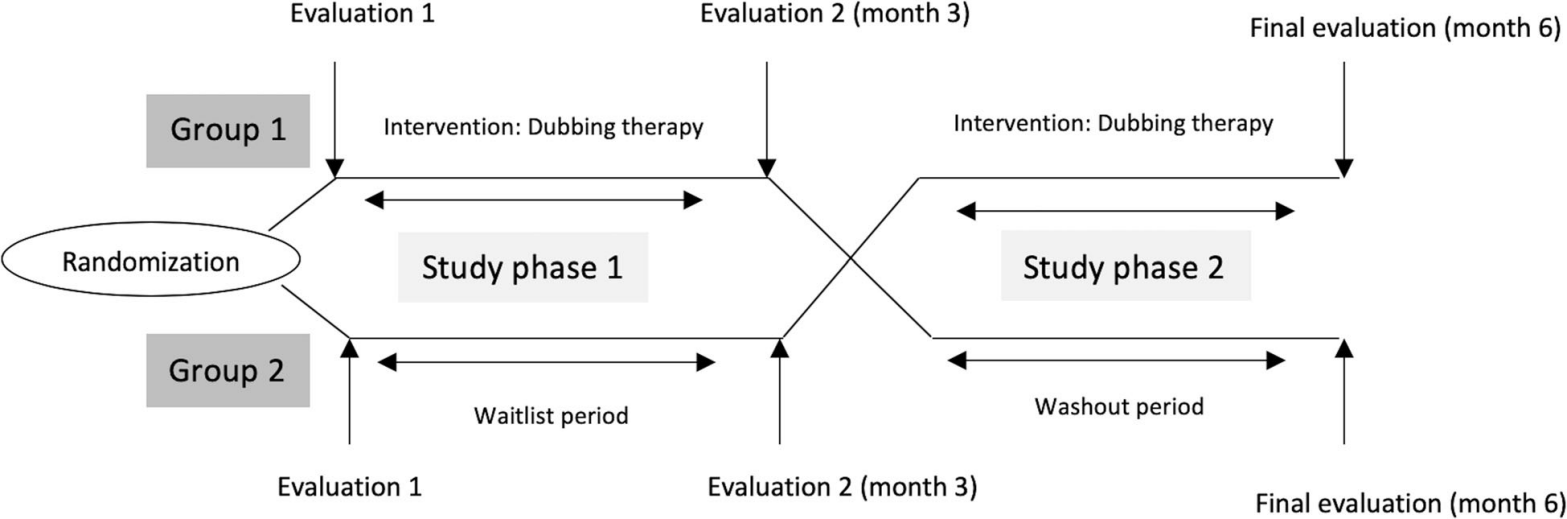
Study design

Upon signature of informed consent, the included patients will be randomly allocated (1:1) to one of the following groups (Fig. 1):

- Group 1 (*N*=27): therapy starts within the first 3 months of their inclusion in the study, followed by a subsequent period of 3 months without therapy (washout period), thus serving as controls for the second phase of the study.
- Group 2 (*N*=27): therapy starts between 3 and 6 months after their inclusion in the study. They do not receive speech therapy treatment during the first 3 months, thus serving as controls for the first phase of the study (waitlist controls) and as the active intervention group in the second phase.

A computer-generated random list of numbers provided by an independent statistician will be used for study allocation. The randomization sequence will be created using SAS V.9.4 statistical software (procedure “PROC PLAN”) with a 1:1 allocation. No randomization seed will be specified. The randomization seed will be generated taking the hour of the computer when the program is executed. Randomization will be performed centrally through an Interactive Web Response System in order to conceal the sequence until interventions are assigned (REDCap 8.7.4 - 2021 Vanderbilt University).

### Intervention

- Baseline session: first, evaluation of the baseline patient’s communication skills through BDAE and CAL. Second, to individualize the therapy, the patients and their relatives will select 48 words according to their needs from a list that was constructed by members of stroke and aphasia patients’ associations through an online survey available at https://goo.gl/forms/sxsDH9sicKaFlvas2. Each word has a corresponding 5- to 15-s clip that will be presented during the therapy following the principle of incremental learning (less complex first) using ad hoc designed software very similar to free audio editing software. Finally, the patient will receive training in the basic points of dubbing.
- Sixteen 40-minute individualized dubbing sessions: one clip is played several times with sound. The dubbing actor explains the context and the speech therapist teaches the patient how to imitate the oral movements of the actor. Then, the target word is muted and the patient is encouraged to dub the gap, regardless of the synchrony, although some indicators may signal when the word should be pronounced. After each recording, the patient is rewarded by watching the dubbed fragment as many times as he/she wants to. We will score the following components: repetition, motor coordination, intonation, and synchrony. They will be evaluated at the end of the therapy considering the following ad hoc criteria for each targeted word: repetition (not achieved, achieved with difficulty, achieved); motor coordination (no impairment, mild impairment, moderate impairment, severe impairment); intonation (appropriate, not appropriate); and synchrony (appropriate, not appropriate).

### Primary outcomes

A psychologist blinded to the patients’ clinical characteristics and his/her allocated group will administer the CAL [28] questionnaire and the BDAE [29] three times per patient. In brief, group 1 will be evaluated after the study group allocation, after the completion of the therapy, and after a washout period of 3 months, whereas group 2 will be evaluated after the study group allocation, after the waitlist period/prior the start of the therapy and after its completion.

### Secondary outcomes

We will include additional tests following the ROMA consensus statement [30] for aphasia treatment research: General Health Questionnaire (GHQ)-12, Stroke Aphasia Quality of Life Scale (SAQOL-39), and the Western Aphasia Battery Revised (WAB-R). In addition, we will include the Stroke Aphasic Depression Questionnaire (SADQ10) to detect depressed mood and we will analyze the number of dropouts. An amendment to the protocol (protocol version 2.0; dated 7 October 2020), which was approved by the ethics committee prior the start of the recruitment, included a substudy to evaluate idiosyncratic and generalization effects with an ancillary multiple-baseline design [31]. We will randomly select 10 participants from the larger group of participants. The single-subject analysis will be comprised of three phases: baseline, treatment, and post-test. The assessor will be blind to the phase status of the participants. Generalization will be assessed by presenting 10-trial blocks of untrained words, untrained words in context, and trained words in context. In the untrained-word trials, the examiner will present a word that had not been used during treatment and will ask the participant to repeat it (e.g., please, repeat “lights”). In the untrained-word-in-context trials, the examiner will present a fill-in-the-blank phrase for target words that had not been used during treatment. Lastly, in the trained-word-in-context trials, the examiner will present a fill-in-the-blank phrase for target words previously used during treatment. We will establish the effect of the intervention for the three types of words (i.e., untrained, untrained in context, trained in context) using the Hedges-Pustejovsky-Shadish model [32]. Effect sizes will be calculated for all two-term comparisons (i.e., baseline vs. treatment, baseline vs. post-test, and treatment vs. post-test). To obtain inter-observer agreement, we will record the audio of 20% of the baseline, treatment, and post-test sessions. An agreement is defined as two independent observers recording the same trial outcome (e.g., correct, incorrect, no response). Interobserver agreement will be computed as the number of trials with agreement divided by the total number of trials multiplied by 100.

### Data management and monitoring body

Data will be prospectively included in a study-specific database developed with REDcap software (REDCap 8.7.4 - 2021 Vanderbilt University). All data management will follow the principles of the European regulations for biomedical research ensuring confidentiality. In compliance with European regulations/International Conference of Harmonization Good Clinical Practice Guidelines, the investigator and the institution are required to permit direct access to authorized representatives of the Ethics Committee to review the subject’s original medical records for verification of study-related procedures and data. Monitoring will be conducted by dedicated personnel at the Clinical Trial Unit at La Paz University Hospital.

### Sample size estimates

A formal sample size calculation is not possible given that this is a pilot study on a new therapy. However, based on a previous feasibility clinical trial on a different SLT in patients with post-stroke aphasia developed by our group [33], we estimated that we would need a sample size of 27 patients in each arm for an 80% power and a 0.050 two-sided significance level to detect a significant effect on the CAL evaluation [28]. Following the CONSORT guidelines for randomized pilot and feasibility trials, [26] upon the completion of this pilot trial we will estimate the sample size calculation for a definitive trial to evaluate the efficacy of this new therapy.

To achieve adequate participant enrolment to reach the target sample size, the following strategies will be implemented: stroke patients admitted to the Stroke Unit of La Paz University Hospital with post-stroke aphasia will be followed up at least 3 months after their stroke and, after the completion of the standard SLT, will be invited to participate in this study. Moreover, we will retrospectively review the clinical charts of patients discharged from the Speech Therapy Unit at La Paz University Hospital during the year before the start of the study and invite them also to participate. Finally, we will provide leaflets with the relevant study information to stroke patients’ associations. The cross-over design allows this experimental therapy to be offered to all the patients who participate in the trial, therefore reducing the possible rate of refusal to be enrolled in the control group. To ensure the follow-up, we will adapt the schedules of the dubbing sessions to the needs of the patients and their relatives.

### Statistical analyses

We will use R software [34]. To evaluate the benefit of DULCINEA therapy through the CAL and the BDAE questionnaires, we will use mixed effects linear regression models, a statistical model particularly useful for longitudinal studies with repeated measures. Because of their advantage in dealing with missing values, mixed effects models are usually preferred over other approaches, allowing for an adjustment of the treatment effects by the baseline values and the period effect in crossover trials. Given this study has a crossover design with two treatment sequences (treatment-washout/waitlist-treatment) and two phases (phase 1 and phase 2) with a baseline evaluation of all the patients, we will consider treatment and phase as principal fixed effects, the treatment*phase as interaction effect and the patient nested in the treatment sequence as random effect. For pairwise post hoc comparisons, we will use the Bonferroni test. Analysis will also look for significant differences within test scores and variables scored for each dubbing session.

## Discussion

Nowadays, one of the major challenges in SLT is to find a standardized, rapid, and low-cost therapy due to the increasing prevalence of stroke survivors; it is estimated that approximately 15–42% of these survivors experience post-stroke aphasia [3]. Although it has been clearly shown that SLT is beneficial for post-stroke aphasia in terms of improved functional communication, reading, writing and expressive language compared with no therapy, the current provision of SLT is heterogeneous with disparities related to the therapy regimen, delivery models, and the treatment setting [7].

In this challenging scenario, the development of an innovative therapy aimed at improving recovery from stroke as well as quality of life of stroke survivors and their relatives would represent an important advance in this field and would help to improve citizens’ health and wellbeing. In addition, computer programmes and apps to continue therapy at home, shown to improve speech output and narrative production in non-controlled studies [12, 13], could save precious time and money for society, patients, and relatives.

Using audiovisual content showing lip movements, on which dubbing techniques are based, and encouraging both healthy controls and post-stroke aphasic patients to imitate them is proven to activate a network of cortical areas involved in planning and executing speech production as demonstrated in functional MRI studies [35–37]. Therefore, there is a well-known neurobiological basis to support the effects on brain connectivity that DULCINEA therapy may produce. According to our preliminary results [25], this novel approach also helps to integrate various language requirements (the simultaneous practice of coordination of oral muscles, rhythm, and emotional intonation); to increase sustained attention and motivation, thus reducing dropouts and to improve functional language (as words are practiced within realistic frameworks, namely, the film scene). Compared with other trials on computer-based SLT, the DULCINEA trial allows the patient and their relatives to choose the target words to be dubbed, according to their needs and preferences. However, given that it is a novel therapeutic approach to post-stroke aphasia, it is unknown whether the potential improvement in functional communication is long-lasting or how long the effects last. This was one of the reasons for the choice of a cross-over design, to evaluate whether the effects last at least 3 months after the intervention. We chose this time point because it was a feasible washout period in our prior feasibility trial on aphasia treatment and it is a longer period than in other trials on aphasia, which lasted only 6 weeks [38, 39]. A 3-month period will give us a better approach to establishing the stability of the potential improvements.

Finally, it has been reported that the relevance and translation of research findings may be increased by considering research outcomes which are important to people living with aphasia [40]. In this sense, we incorporated from the very beginning the voice of patients with aphasia and their relatives, who contribute to the study design, mainly in the selection of words to be dubbed, and in the educational and dissemination activities scheduled in the DULCINEA project.

## Conclusions

This pilot clinical trial exemplifies the collaboration between hospitals, universities, and patients in the development of a new therapeutic tool based on dubbing techniques in our aim to improve the recovery and quality of life of aphasic patients based on functional communication, computer-based technology, and standardized test assessment.

## Trial status

Recruitment started in December 2020, and it is planned to end by March 2023. Protocol version 1.0, date 15 July 2019; Protocol version 2.0, dated October 7, 2020).

## Data Availability

Trial results will be published in Open Access journals. Raw data will be available from the corresponding author upon reasonable request.

## Abbreviations

BDAE: Boston Diagnostic Aphasia Examination
CAL: Communicative Activity Log questionnaire
DULCINEA: DUbbing Language-therapy CINEma-based in Aphasia post-Stroke
GHQ-12: General Health Questionnaire
SADQ10: Stroke Aphasic Depression Questionnaire
SAQOL-39: Stroke Aphasia Quality of Life Scale
SLT: Speech and language therapy
WAB-R: Western Aphasia Battery Revised
WP: Work package
